# Efficacy and safety of dolutegravir plus lamivudine for patients with late presentation of HIV-1 infection: a retrospective real-world cohort study in Southwest China

**DOI:** 10.3389/fmed.2025.1630960

**Published:** 2025-08-29

**Authors:** Qing Wang, Xiaoxin Xie, Yanhua Fu, Xiaoyan Yang, Lin Gan, Shujing Ma, Pengzhu Jiao, Muli Wu, Jun Li, Hai Long

**Affiliations:** ^1^School of Public Health, The Key Laboratory of Environmental Pollution Monitoring and Disease Control, Ministry of Education, Guizhou Medical University, Guiyang, Guizhou, China; ^2^Department of Infection, Guiyang Public Health Clinical Center, Guiyang, Guizhou, China

**Keywords:** human immunodeficiency virus 1, efficacy, dolutegravir plus lamivudine, late presentation, retrospective real-world cohort study

## Abstract

**Background:**

Evidence regarding the use of dolutegravir plus lamivudine (DTG + 3TC) among patients with HIV infection who present late remains limited. This study aimed to evaluate the effectiveness and safety of DTG + 3TC therapy in patients with late presentation in Southwest China.

**Methods:**

This single-center, retrospective cohort study included patients with late presentation who initiated DTG + 3TC anti-retroviral therapy (ART) between January 2020 and July 2023 (*N* = 176). Changes in immunologic and metabolic parameters as well as liver and kidney function, were assessed. The primary endpoint was the proportion of participants with HIV-1 RNA < 50 copies/mL at week 48. Late presentation was defined as CD4 < 350 cells/μL or the presence of AIDS-defining conditions.

**Results:**

At weeks 24 and 48, 83.0% (146/176) and 90.9% (160/176) of the patients achieved HIV-1 RNA levels <50 copies/mL, respectively. At week 48, the median CD4 count increased by 139.5 cells/μL (120.5–158.5), and the CD4/CD8 ratio increased by 0.2 (0.1–0.3) (*p* < 0.001). No patient discontinued treatment owing to adverse events during the observation period.

**Conclusion:**

DTG + 3TC demonstrated high virologic efficacy and good tolerability in patients with late presentation. However, the regimen may be associated with an increase in lipid levels and weight, highlighting the need for regular monitoring.

## Introduction

People diagnosed late with HIV-1 infection are a vulnerable group and experience delayed diagnosis and treatment. Late presentation hinders epidemic control and is associated with high mortality rates (1), high transmission (2), and high treatment costs, all of which pose major challenges in global human immunodeficiency virus (HIV) prevention and control (3). Unfortunately, even with high-cost treatment, the health status of patients with late presentation remains worse than that of those who seek timely care, and their medical expenses continue to rise, placing significant burdens on both the individual patients and healthcare system (3). Untreated late presentation is a common issue in global HIV prevention and control. For instance, a study encompassing 17 European countries demonstrated that the late-presenting rate was 48.4% (4), with China’s total late-presenting rate at 43.26% between 2010 and 2020 (5). These data suggest that late presentation is one of the major challenges facing the global context of acquired immunodeficiency syndrome (AIDS).

Anti-retroviral therapy (ART) transforms AIDS from a fatal disease into a manageable chronic condition (6). Since there is no cure for AIDS, patients need to take medication for life. Long-term drug use may lead to drug toxicities, such as cardiovascular disease (7), liver and kidney damage (8, 9), and neurocognitive disorders (10). These drugs can seriously affect the quality of life of people living with HIV (PLWH). The elderly (≥50 years) are the most important high-risk group for late presentation in China (5). With universal access to antiretroviral treatment, more than 20% patients with late presentation worldwide are over 50 years old (11). Considering that the numbers of associated chronic comorbidities and medications increase with age (12), the combination of ART and non-ART medications can further exacerbate drug toxicities in this patient population. Therefore, suitable treatment options are needed for patients aged over 50 years with late presentation.

A standard ART initiation regimen typically comprises a three-drug combination (13–16). To reduce drug toxicity and drug–drug interactions, two-drug regimens (2DRs) have become a focus of recent research, with the aim of lowering the overall drug burden while maintaining an efficacy comparable to that of three-drug regimens (3DRs). Second-generation integrase strand transfer inhibitors (INSTIs), such as dolutegravir (DTG), are considered ideal core agents because of their high barrier to resistance, strong efficacy, favorable safety profile, and minimal drug–drug interactions (17). Currently, multiple international and Chinese guidelines recommend DTG + lamivudine (3TC) as a first-line regimen for the treatment of PLWH (13–16). Numerous studies have demonstrated the efficacy and safety of DTG + 3TC as an initial regimen (18–21), and others have shown that DTG + 3TC is non-inferior to 3DRs in treatment-naïve PLWH (20–23).

Theoretically, ART should be initiated in patients with late presentation as soon as possible, even before resistance test results and other test results are available. Therefore, the optimal regimen should have high efficacy and a high barrier to resistance, and 2DRs of DTG + 3TC may be a cost-effective and efficient option for those with late presentation. This study aimed to evaluate the efficacy and safety of the DTG + 3TC regimen in patients with late HIV presentation in Southwest China.

## Materials and methods

### Study design and participants

This was an observational, single-center, retrospective study. The study site was the Guiyang Public Health Clinical Center, which manages approximately 20% of the PLWH in Guizhou Province, China, and is one of the largest infectious disease hospitals in Southwest China. Newly reported cases of late presentation between 1 January 2020 and 31 July 2023 were recruited as the study population and followed until 31 July 2024. The inclusion criteria were as follows: (1) HIV-1 antibody confirmed positive by Western blot, (2) age ≥18 years, (3) treatment-naïve, late presentation of HIV-1 infection, (4) and ART regimen of DTG + 3TC [including DTG/3TC (compound single tablet preparation) or DTG + 3TC (two pills) simplified double regimen, including patients with 3TC reduction due to abnormal renal function]. The exclusion criteria were as follows: (1) presence of hepatitis B virus (HBV) infection, (2) pregnancy, (3) loss to follow-up or death, and (4) incomplete baseline information.

### Study endpoints

The primary endpoint was the rate of virologic suppression after 48 weeks on the DTG + 3TC regimen, calculated as the proportion of patients with HIV-1 RNA < 50 copies/mL. The secondary endpoints were as follows: (1) virologic efficacy after 24 weeks with the DTG + 3TC regimen and (2) changes in immunologic efficacy (CD4, CD4/CD8), metabolic function, liver and kidney function and occurrence of adverse events (AEs) at week 48.

### Definitions

According to international and Chinese guidelines, late presentation was defined as CD4 < 350 cells/μL or meeting the criteria for AIDS-defining conditions (4, 16, 24, 25). AIDS-defining conditions were as follows (16): persistent irregular fever of unknown cause, diarrhea for >1 month (>3 bowel movements/day), decrease in body mass >10% within 1 month, recurrent oral fungal infections, recurrent herpes simplex virus infection or herpes zoster virus infection, *Pneumocystis jirovecii* pneumonia (PJP), recurrent bacterial pneumonia, active tuberculosis (TB) or nontuberculosis mycobacteria disease, deep fungal infection, central nervous system space-occupying lesions, development of dementia as a middle-aged or young person, active cytomegalovirus infection, *Toxoplasma gondii* encephalopathy, *Penicillium marneffei* infection, recurrent sepsis, Kaposi sarcoma, and lymphoma.

Virologic suppression was defined as HIV-1 RNA < 50 copies/mL at week 48. Virologic failure was defined as HIV-1 RNA ≥ 200 copies/mL at week 48. When ART was initiated within 7 days of HIV-1 antibody confirmation, it was considered as rapid ART.

Dyslipidemia was defined as follows: total cholesterol (TC) ≥ 5.2 mmol/L, triglyceride (TG) level ≥1.7 mmol/L, high-density lipoprotein cholesterol (HDL-C) < 1 mmol/L, and low-density lipoprotein cholesterol (LDL-C) ≥ 3.4 mmol/L (26).

Abnormal liver function was defined as aspartate aminotransferase (AST) > 40 U/L and alanine aminotransferase (ALT) level >50 U/L (27).

Renal dysfunction was defined as estimated glomerular filtration rate (eGFR) < 60 mL/min (28, 29). The eGFR is calculated using the CKD-EPI formula, which includes sex, age, and serum creatinine (Scr), and can represent changes in renal function in a more comprehensive way (30).

### Data collection and laboratory tests

Participants’ information was obtained from the China AIDS Prevention and Control Information System and the case system of the Guiyang Public Health Clinical Center. The following information was collected at baseline and weeks 24 and 48: (1) basic personal information: age, sex, infection characteristics, height, weight, date of HIV-1 diagnosis, date of ART initiation; (2) laboratory test results: HIV-1 RNA, CD4 count, complete blood count, lipid levels (four items), glucose (GLU), and amylase (AMY); and (3) other information: comorbidities (clinical diagnosis), AIDS-defining conditions (clinical diagnosis), primary reasons for use of DTG + 3TC, and AEs. To ensure data comparability, all tests were performed at the Guiyang Public Health Clinical Center, using standardized instruments, reagents, and methodologies. Participants with missing baseline data were excluded. For participants lacking data at the 24- and 48-week time points, the following procedures were applied: (1) results within the 30-day windows of the target time points were accepted; (2) if no window data were available, direct imputation was performed when missingness was <5%. The imputation method was based on the Kolmogorov–Smirnov test: mean imputation was applied to normally distributed data (*p* > 0.05), and median imputation, to non-normally distributed data.

### Statistical analysis

Excel was used to input data, and R 4.3.1 was used for statistical analysis. Based on the data distribution type, qualitative variables were reported as frequency distributions, whereas quantitative variables were described as median (IQR) or mean (SD). The Kolmogorov–Smirnov test was used to determine whether the numerical variables fit the assumptions for normality of distribution. Paired t-test was used to compare normally distributed independent variables between the baseline and week 48, while Wilcoxon signed-rank test was used for continuous numeric variables that followed a non-normal distribution. Classification data were compared using the chi-square or Fisher’s exact tests. All statistical tests were two-tailed, and *p* < 0.05 was considered statistically significant.

## Results

### Patient characteristics

**Figure 1:**
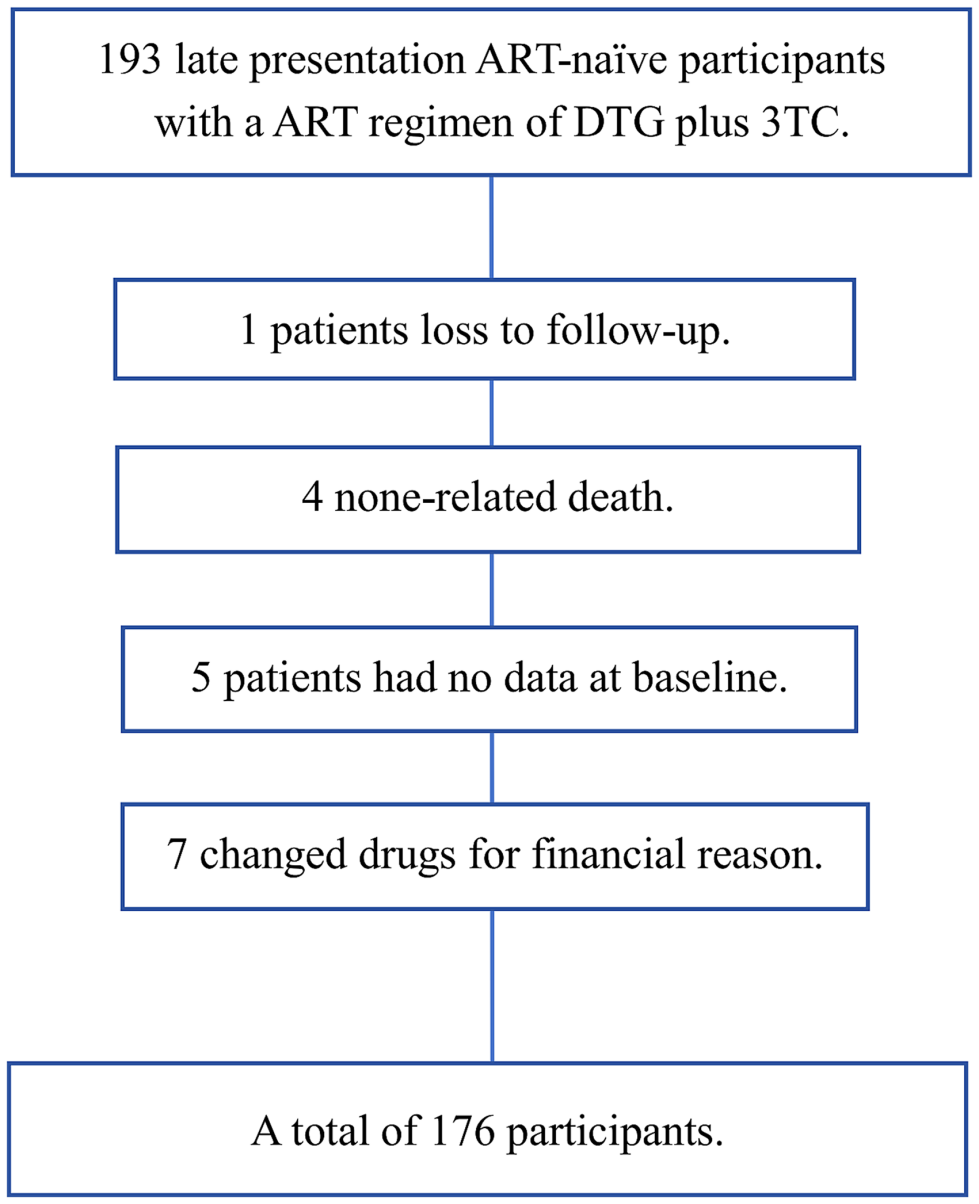
Participant selection process DTG + 3TC, dolutegravir plus lamivudine, including DTG/3TC (compound single tablet preparation) or DTG + 3TC (two pills) simplified double regimen.

The patient selection process is shown in Figure 1. A total of 193 patients received DTG + 3TC between January 2020 and July 2023. Among these patients, one patient was lost to follow-up, four patients died (unrelated to the medication), five patients had no baseline data, and seven patients changed medications due to financial reasons. Finally, 176 participants were included.

The baseline characteristics of the patients are described in Table 1. The patients were predominantly male (*n* = 132, 75.0%). The median age of the patients was 63.5 (52.0–72.0) years, and the proportion of older patients (≥50 years) was as high as 76.7% (135/176). At baseline, the median CD4 count was 144.5 (54.3–204.8) cells/μL, with 128 (72.7%) patients having a CD4 count of <200 cells/μL. The median log10 HIV-1 RNA was 5.0 (4.5–5.5) copies/mL; 31 (17.6%) patients had HIV-1 RNA ≥ 5 × 10^5^ copies/mL; 29.0% (51/176) of the patients received rapid ART. In our patient group, 140 (79.5%) patients had comorbidities, the most common being renal impairment, at 35.2% (62/176), followed by dyslipidemia (*n* = 59, 33.5%), cardiovascular disease (CVD) (*n* = 49, 27.8%), liver impairment (*n* = 41, 23.3%), hypertension (*n* = 36, 20.5%), diabetes (*n* = 21, 11.9%), and cancer (*n* = 10, 5.7%). Among all AIDS-defining conditions (*n* = 78, 44.3%), the most common was invasive fungal disease (*n* = 54, 30.7%), followed by PJP (*n* = 30, 17.0%), candidiasis (*n* = 26, 14.8%), and TB (*n* = 24, 13.6%). The main reasons for choosing DTG + 3TC included low potential for drug–drug interaction (*n* = 93, 52.8%), renal impairment (*n* = 44, 25.0%), and ease of use (*n* = 36, 20.5%).

**Table 1:** Baseline demographic and clinical characteristics.

Characteristic	DTG + 3TC (*N* = 176)
Sex^a^	
Male	132 (75.0)
Female	44 (25.0)
Age^b^ (year)	63.5 (52.0–72.0)
≥50 years^a^	135 (76.7)
Infection characteristics^a^	
Heterosexual transmission	164 (93.2)
Homosexual transmission	12 (6.8)
BMI^b^ (kg/m^2^)	20.6 (18.7–22.5)
CD4 count^b^ (cells/μL)	144.5 (54.3–204.8)
<200^a^	128 (72.7)
200–349^a^	40 (22.7)
≥350^a^	8 (4.6)
HIV-1 RNA, log10^b^ (copies/mL)	5.0 (4.5–5.5)
<100,000^a^	93 (52.9)
100,000–499,999^a^	52 (29.5)
≥500,000^a^	31 (17.6)
Initiation of ART^a^	
Rapid ART	51 (29.0)
Non-Rapid ART	125 (71.0)
Comorbidities^a^	140 (79.5)
CVD	49 (27.8)
Hypertension	36 (20.5)
Diabetes	21 (11.9)
Dyslipidemia	59 (33.5)
Renal impairment	62 (35.2)
Liver impairment	41 (23.3)
Cancer	10 (5.7)
Herpes Zoster	13 (7.4)
Syphilis	21 (11.9)
AIDS-defining conditions^a^	78 (44.3)
Invasive fungal disease	54 (30.7)
PJP	30 (17.0)
Candidiasis	26 (14.8)
TB	24 (13.6)
*Penicillium marneffei* infection	12 (6.8)
Cryptococcosis	10 (5.7)
Active cytomegalovirus infection	6 (3.4)
*Toxoplasma gondii* encephalopathy	4 (2.3)
Primary reasons for use of DTG + 3TC^a^	
Low potential for drug–drug interaction	93 (52.8)
Renal impairment	44 (25.0)
Easy to take	36 (20.5)
Dyslipidemia	32 (18.2)
Liver impairment	22 (12.5)

^a^, N (%); IQR, interquartile range; b, Median (IQR); DTG + 3TC, dolutegravir plus lamivudine, including DTG/3TC (compound single tablet preparation) or DTG+3TC (two pills) simplified double regimen; ART, Anti-retroviral therapy; Rapid ART, ART initiated within 7 days of HIV-1 antibody confirmation; AIDS, acquired immunodeficiency syndrome; CVD, Cardiovascular disease; PJP, *Pneumocystis jirovecii* pneumonia; TB, tuberculosis.

### Virologic efficacy

**Figure 2:**
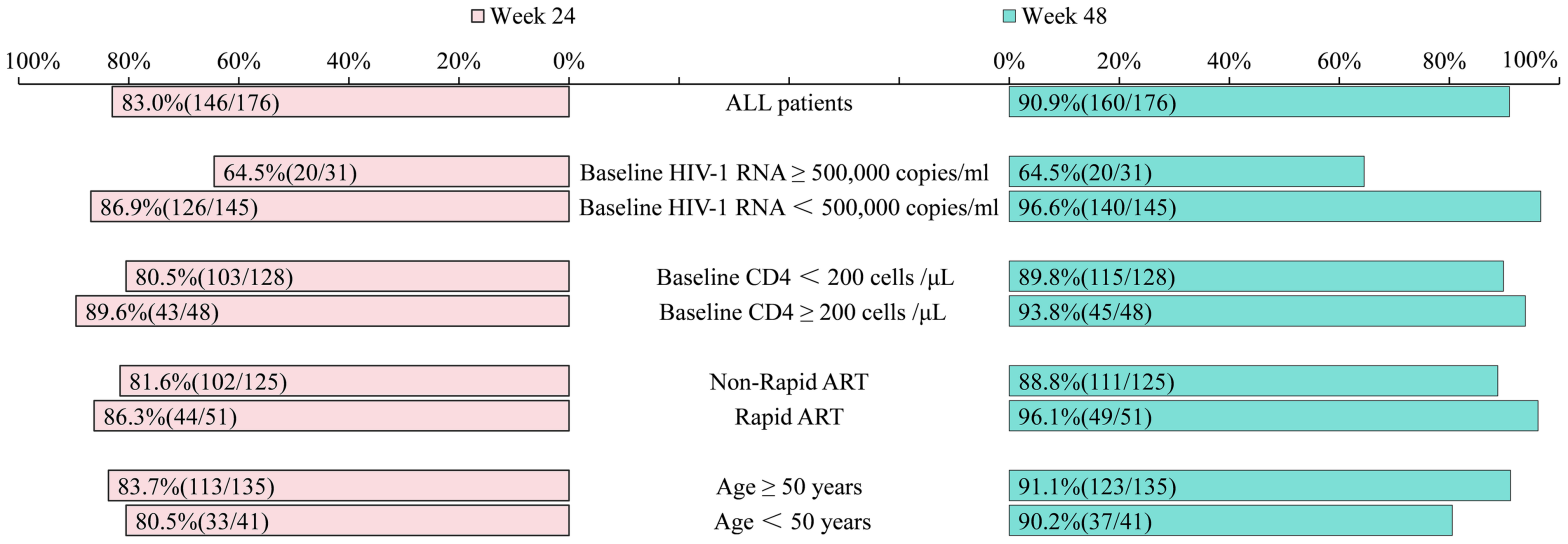
Comparison of the proportion of patients with HIV-1 RNA < 50 copies/mL at week 24 versus week 48 (%). The same standardized instruments, reagents, and methodologies were used throughout the process to detect viral load. ART, Anti-retroviral therapy; Rapid ART, ART initiated within 7 days of HIV-1 antibody confirmation.

At week 24, 83.0% (146/176) of the participants had HIV-1 RNA levels <50 copies/mL, while 4.5% (8/176) had levels ≥200 copies/mL. The virologic suppression rates were 64.5% (20/31) for those with baseline HIV-1 RNA ≥ 500,000 copies/mL and 86.9% (126/145) for those with HIV-1 RNA < 500,000 copies/mL. Participants with baseline CD4 counts <200 cells/μL had a virologic suppression rate of 80.5% (103/128), compared to 89.6% (43/48) for those with CD4 counts ≥200 cells/μL. Suppression rates for rapid and non-rapid ART initiation were 86.3% (44/51) and 81.6% (102/125), respectively. Among participants aged ≥50 years and <50 years, the suppression rates were 83.7% (113/135) and 80.5% (33/41), respectively (Figure 2).

At week 48, 90.9% (160/176) of the participants had HIV-1 RNA levels <50 copies/mL, and an intention to treat (snapshot) analysis showed that if all 193 patients who ever received DTG + 3TC were retained and the 17 now excluded (losses, deaths, baseline data gaps, economic switches) were counted as non-suppressed, the viral suppression rate at week 48 would be 160 /193 ≈ 83.0%, not 90.9% (160/176). 3.4% (6/176) of the participants would have RNA levels of ≥200 copies/mL. The virologic suppression rates were 64.5% (20/31) and 96.6% (140/145) for those with baseline HIV-1 RNA ≥ 500,000 and <500,000 copies/mL, respectively. The suppression rates for baseline CD4 < 200 cells/μL and CD4 ≥ 200 cells/μL were 89.8% (115/128) and 93.8% (45/48), respectively. Rapid ART initiation resulted in a suppression rate of 96.1% (49/51), compared to 88.8% (111/125) for non-rapid initiation. Among participants aged ≥50 years and <50 years, the suppression rates at baseline were 91.1% (123/135) and 90.2% (37/41), respectively (Figure 2).

At week 48, 16 participants in the study had HIV-1 RNA ≥ 50 copies/mL. Among them, eight had documented treatment discontinuation, and 11 achieved virologic suppression during subsequent follow-up. Notably, in one patient with a 48-week HIV-1 RNA level of 913,000 copies/mL, who switched to a 3-drug bictegravir (BIC) regimen, the level declined to 54 copies/mL after 32 weeks. Three patients had no available viral load (VL) test results after week 48. At week 48, 11 patients with baseline HIV-1 RNA ≥ 500,000 copies/mL had HIV-1 RNA levels ≥50 copies/mL, of whom five exhibited treatment discontinuation. Among patients with baseline CD4 counts <200 cells/μL, 13 had HIV-1 RNA ≥ 50 copies/mL, and six of these had self-discontinuation behavior. Of the 16 virologically unsuppressed patients, seven underwent baseline resistance testing, and one was found to have resistance at locus V179E (Table 2).

**Table 2:** Characteristics of individuals with ≥50 copies/mL of HIV-1 RNA after 48 weeks.

				HIV-1 RNA (copies/mL)				CD4 (cells/μL)				
Number	Age, year	Sex	Comorbidities/AIDS-defining conditions	Baseline	Week 24	Week 48	Other records HIV-1 RNA	Baseline	Week 24	Week 48	Skipping Pills, day	Drug resistance
1	76	Male	TB, Renal impairment, diabetes, dyslipidemia	698,000	0	58	at week 96; 22.9, at week 144	161	277	328	0	NA
2	76	Male	NA	545,000	0	42,400	NA	24	219	136	60	NA
3	68	Male	Renal impairment, dyslipidemia	10,000,000	0	108	TND, at week 144	310	652	652	60	NA
4	91	Male	Renal impairment, dyslipidemia, diabetes, hypertension	839,000	<20	51	TND, at week 36 and 72	147	212	245	0	NA
5	57	Male	CVD, dyslipidemia, PJP	1,540,000	<20	284,000	34.6, at week 72	55	221	242	0	NO
6	44	Male	PJP, Candidiasis	946,000	136	1,670	38.5, at week 72	35	136	169	0	NO
7	47	Female	NA	1,550,000	51,600	401	<20, at week 96	85	176	296	16	NA
8	68	Male	Renal impairment, cryptococcosis	10,000,000	30,000	108	36.3, at week 96	22	297	192	120	NO
9	84	Male	Dyslipidemia, renal impairment	10,000,000	56.8	8,260	NA	117	109	157	60	NA
10	67	Male	Renal impairment	775,000	83	82	NA	249	649	506	0	NA
11	21	Male	Liver impairment, herpes zoster, *Penicillium marneffei* infection	6,090,000	167	81	TND, at week 76	25	142	201	0	NA
12	57	Male	Syphilis, PJP	5,850	0	174	TND, at week 84	48	208	178	10	NO
13	65	Male	Dyslipidemia	299,000	169	97	42.4, at week 96	344	566	506	50	NA
14	73	Male	Hypertension	451,000	137	157	129, at week 88	173	411	495	0	NO
15	56	Male	Dyslipidemia, renal impairment, PJP	272,000	65.1	913,000	54, 32 weeks after changing BIC drug regimen	25	159	173	30	NO
16	26	Male	Liver impairment, candidiasis	287,000	399	126	TND, at week 72	5	56	68	0	V179E

NA, not applicable; TND, target not detected, indicating below detection limit; NO, not detected; CVD, cardiovascular disease; PJP, *pneumocystis jirovecii* pneumonia; TB, tuberculosis; BIC, bictegravir.

### Immunologic, metabolic, and organ function outcomes

After 48 weeks, the mean CD4 count increased by 139.5 (120.5–158.5) cells/μL, and the CD4/CD8 ratio increased by 0.2 (0.1–0.3) (*p* < 0.001; Table 3). The number of patients with CD4 counts ≥200 cells/μL increased from 49 (27.8%) at baseline to 121 (68.8%) at week 48 (*p* < 0.001, Table 3). Metabolically, significant increments in weight [3.0 (2.5–4.0)]; body mass index [BMI; 1.2 (0.9–1.5)]; and TC [0.5 (0.4–0.7)], TG [0.2 (0.1–0.3)], HDL-C [0.3 (0.2–0.4)], and LDL-C [0.3 (0.1–0.4)] levels were observed. These changes were statistically significant (TG: *p* = 0.022, all others: *p* < 0.001). Regarding liver function, the levels of ALT [−6 (−8.5 to −4.0)], AST [−8.5 (−11.0 to −6.5)], and lactate dehydrogenase [LDH; −41.5 (−54.5 to −32.0)] decreased (*p* < 0.001). In contrast, total bilirubin [TBIL; 2.1 (1.3–2.8)], direct bilirubin [DBIL; 0.8 (0.5–1.1)], and indirect bilirubin [IBIL; 1.3 (0.7–1.9)] levels increased (*p* < 0.001), although these changes were not clinically significant. In terms of renal function, Scr [19.5 (16.3–22.8)] and uric acid levels [UA; 31.0 (14.5–46.0)] increased (*p* < 0.001), while eGFR level [−8.6 (−11.8 to −5.2)] decreased (*p* < 0.001). Urea levels did not change significantly (Table 3).

**Table 3:** Changes in immunological response and biomarkers at week 48.

Biomarkers	Mean (SD)/Median (IQR)/N (%)		Change from baseline to week 48 (95% CI)	*p* value
	Baseline	Week 48		
CD4 count ^a^ (cells/μL)	144.5 (54.3–204.8)	266.5 (174.3–403.0)	139.5 (120.5–158.5)	< 0.001
CD4 count ^b^ ≥ 200	49 (27.8)	121 (68.8)	NA	< 0.001
CD4/CD8^a^	0.22 (0.13–0.36)	0.46 (0.24–0.72)	0.2 (0.1–0.3)	< 0.001
Weight^a^ (kg)	55.0 (50.0–60.0)	57.2 (52.0–63.8)	3.0 (2.5–4.0)	< 0.001
BMI^a^ (kg/m^2^)	20.6 (18.7–22.5)	21.6 (19.7–24.2)	1.2 (0.9–1.5)	< 0.001
TC^a^ (mmol/L)	3.9 (3.2–4.6)	4.3 (3.7–5.2)	0.5 (0.4–0.7)	< 0.001
TG^a^ (mmol/L)	1.5 (1.1–2.1)	1.7 (1.2–2.4)	0.2 (0.1–0.3)	0.022
HDL-C^a^ (mmol/L)	0.8 (0.6–1.1)	1.1 (0.9–1.3)	0.3 (0.2–0.4)	< 0.001
LDL-C^c^ (mmol/L)	2.3 (0.7)	2.5 (0.9)	0.3 (0.1–0.4)	< 0.001
ALT^a^ (U/L)	21.0 (14.0–34.0)	16.0 (12.0–23.0)	-6 (−8.5 to −4.0)	< 0.001
AST^a^ (U/L)	29.0 (23.0–41.0)	22.0 (18.0–26.8)	−8.5 (−11.0 to −6.5)	< 0.001
LDH^a^ (U/L)	218.0 (184.0–285.8)	183.0 (161.3–205.0)	−41.5 (−54.5 to −32.0)	< 0.001
TBIL^a^ (μmol/L)	8.7 (6.6–11.6)	10.9 (7.6–14.8)	2.1 (1.3–2.8)	< 0.001
DBIL^a^ (μmol/L)	3.0 (2.2–4.1)	3.9 (3.1–5.1)	0.8 (0.5–1.1)	< 0.001
IBIL^a^ (μmol/L)	5.7 (3.8–7.8)	6.6 (4.2–9.7)	1.3 (0.7–1.9)	< 0.001
Urea^a^ (mmol/L)	5.2 (4.2–7.2)	5.3 (4.2–6.8)	−1.2 (−0.5 to 0.2)	0.324
Scr^a^ (μmol/L)	71.0 (55.0–91.2)	90.0 (76.0–111.9)	19.5 (16.3–22.8)	< 0.001
eGFR^a^(mL/mim)	67.9 (49.1–93.9)	63.2 (42.8–89.7)	−8.6 (−11.8 to −5.2)	< 0.001
UA^a^(μmol/L)	335.0 (254.8–425.3)	368.5 (308.3–445.0)	31.0 (14.5–46.0)	< 0.001
AMY^a^ (U/L)	85.0 (64.3–110.0)	84.0 (65.0–105.8)	−2.5 (−6.5–1.0)	0.146
GLU^a^ (mmol/L)	5.7 (5.0–6.9)	5.4 (5.0–6.5)	−0.2 (−0.4–0.1)	0.113

NA, not applicable; a, Median (IQR); b, N (%); c, Mean (SD); IQR, interquartile range; CI, confidence interval; BMI: body mass index; Lipid profile (TC, total cholesterol; TG, triglycerides; HDL-C, high-density lipoprotein cholesterol; LDL-C, low-density lipoprotein cholesterol); Liver markers (ALT, alanine aminotransferase; AST, aspartate aminotransferase; LDH, lactate dehydrogenase; TBIL, total bilirubin; DBIL, direct bilirubin; IBIL, indirect bilirubin) Renal markers (Scr, serum creatinine; eGFR, estimated glomerular filtration rate; UA, uric acid); AMY, amylase; GLU, glucose.

### Adverse events

AEs occurred in 27 (15.3%) patients. Among them, seven (4.0%) patients were hospitalized for the following reasons: pulmonary issues (two cases), myocardial infarction, lung cancer, gastrointestinal bleeding, cytomegalovirus retinopathy, and osteophyte formation. Drug-related AEs occurred in five (2.8%) patients: nausea in one (0.6%) patient and diarrhea in four (2.3%). During the treatment period, no patient discontinued the medication owing to lack of drug efficacy or adverse effects (Table 4).

**Table 4:** Occurrence of adverse events.

Adverse event	N (%)
Any	
Arthralgia	2 (1.1)
Bellyache	1 (0.6)
Anxiety	1 (0.6)
Insomnia	2 (1.1)
Serious (hospitalization)	7 (4.0)
Pruritus	6 (3.4)
Herpes zoster infection	5 (2.8)
Drug related	
Nausea	1 (0.6)
Diarrhea	4 (2.3)

## Discussion

This is the first retrospective cohort study in China to evaluate the efficacy and safety of DTG + 3TC in late-presenting individuals with HIV in a real-world setting. Virologic suppression rates were high at both 24 and 48 weeks, and no patient discontinued DTG + 3TC owing to adverse events.

At 24 weeks, the virologic suppression rate in the late-presentation cohort was 83.0% (146/176), which is lower than that reported in the STAT study (18). At week 48, the suppression rate increased to 90.9%, slightly below the rates observed in the GEMINI 1 and 2 trials (21–23). These discrepancies may be explained by the demographic and clinical characteristics of our cohort, including older age and low CD4 counts. Furthermore, patients with late presentation often experience greater challenges with medication adherence (31). In our study, among the 16 patients without virologic suppression, eight exhibited self-discontinuation of treatment for durations ranging from 10 to 120 days. Thus, non-adherence significantly impacted virologic outcomes, underscoring the importance of adherence education for individuals with late presentation of HIV.

Although DTG + 3TC is not currently recommended for treatment-naïve individuals with HIV-1 RNA ≥ 500,000 copies/mL (13–16), emerging evidence supports its use in this subgroup. The STAT study demonstrated suppression rates of 68% at week 24 and 89% at week 48 (18). In a study by Dou et al., suppression rates increased from 50% at 24 weeks to 78.3% at 48 weeks (20). In this study, virologic suppression reached 64.5% (20/31) at week 24, which was higher than that reported by Dou et al. (20) and lower than the rate observed in the STAT study (18), likely because high baseline viral loads are associated with delayed suppression (32). However, the suppression rate at week 48 remained at 64.5% (20/31), still lower than that reported in both the STAT study (18) and the study by Dou et al. (20). Notably, two of the five individuals with high baseline viral loads who achieved suppression at week 24 subsequently discontinued therapy and experienced viral rebound at week 48, suggesting that nonadherence may have contributed to the suboptimal outcomes. Whether DTG + 3TC is effective in reducing viral load in PLWH who present late with high viral burdens remains unclear and warrants further research.

The INSIGHT START study recently confirmed that early detection of HIV and timely initiation of ART are optimal strategies for preventing immune depletion and maintaining immune balance, thereby significantly lowering HIV-related morbidity and mortality (33). In our study, the patients who initiated ART within 7 days achieved a virologic suppression rate of 86.3% at week 24, which increased to 96.1% at week 48—findings consistent with those reported by Gan (34). These results suggest that rapid ART initiation in patients with late presentation not only enhances virologic control but also reduces the risk of HIV transmission.

As the prevalence of chronic comorbidities and polypharmacy increases with age, simplified ART regimens offer several advantages for older adults, including reduced drug–drug interactions, low cumulative toxicity, and improved tolerability and safety profiles. In our study, virologic suppression in patients aged ≥50 years increased from 83.7% (113/135) at week 24 to 91.1% (123/135) at week 48. These findings support the use of DTG + 3TC as an optimized option for late-presenting older adults with HIV.

The CD4 count and CD4/CD8 ratio are key indicators of immune system function. A reduced CD4/CD8 ratio reflects immune dysregulation and increased systemic inflammation (35) and is associated with a greater risk of AIDS-defining conditions and all-cause mortality (36). In our study, patients with baseline CD4 < 200 cells/μL achieved a virologic suppression rate of 89.8% (115/128) at week 48, consistent with the findings of Hou et al. (19). Additionally, after 48 weeks of treatment, both CD4 count and CD4/CD8 ratio increased significantly, with the proportion of patients having CD4 ≥ 200 cells/μL rising from 27.8 to 68.8%. These results indicate substantial immune restoration, aligning with the results of previous studies (18–21).

Long-term ART is known to negatively affect lipid metabolism, and dyslipidemia remains an independent risk factor for cardiovascular disease that requires careful monitoring (37). Current evidence suggests that INSTIs have relatively minor effects on lipid profiles (38). In our cohort, the levels of three lipid (TC, HDL-C, LDL-C) parameters increased by week 48 but remained within the normal reference ranges, consistent with findings from the GEMINI 1 and 2 studies (21) and of Deng et al. (39). Notably, GEMINI 1 and 2 (23) reported no dyslipidemia-related adverse events over a 3-year follow-up period, supporting the lipid-friendly profile of the DTG + 3TC regimen. In the present study, TG levels just reached the threshold for abnormal values (TG ≥ 1.7 mmol/L). TG levels are closely related to diet and lifestyle, and in cases where other lipid levels are normal and TG levels are not significantly elevated, only regular monitoring is required (26). In addition, TG levels tend to increase with age (26), and the median age of the patients in this study was 63.5 years (52.0–72.0), and 76.7% (135/176) of the patients were >50 years old; therefore, the high TG levels could be attributed to the age of our cohort. Based on the above analysis, we concluded that the changes in TG were not clinically significant and did not yet necessitate clinical intervention, but enhanced long-term monitoring of the four lipids is needed to facilitate timely intervention in the event of an abnormality. Similarly, an increase in weight and BMI observed at week 48 was consistent with the results of the GEMINI studies (21) and Wei et al. (40). This finding suggests improved overall health, considering that weight gain in individuals with HIV is often associated with reduced HIV-related inflammation and reversal of catabolic processes (41). Additionally, INSTIs have been shown to directly affect adipose tissue, particularly by inhibiting adipose shrinkage, which can lead to adipofibrosis and hypertrophy, ultimately contributing to weight gain (42). However, the observation period in this study was relatively short, and long-term monitoring of body weight and BMI is needed to further explore the relationships among weight, BMI, and metabolic changes.

DTG has been shown to inhibit organic cation transporter 2 (OCT2), leading to increased Scr levels. This mechanism reflects the non-pathological inhibition of proximal renal tubular secretion, indicating that the renal effects of DTG are reversible and clinically benign (43). After 48 weeks of treatment, a slight increase in Scr and slight decrease in eGFR were observed, consistent with the findings reported by Deng et al. (39). In our study, the patients’ eGFR was within the normal range (eGFR ≥ 60 mL/min) after 48 weeks, and no patient developed severe renal impairment or required regimen changes due to renal toxicity, suggesting that the observed renal function changes were not clinically significant.

This study has some limitations. It was a single-center, region-specific study (in Southwest China), retrospective analysis with a small sample size. Patients who were lost to follow-up, died, lacked baseline data, or switched medications for economic reasons were excluded, and this may have affected the representativeness and generalizability of the findings. Additionally, because resistance testing was cost-prohibitive for many patients, we could not precisely analyze the virologic failure mechanisms. Future studies should include multicenter cohorts, longer follow-up periods, and dedicated patient support funds to improve resistance testing access and ensure broader representativeness.

## Conclusion

The DTG + 3TC regimen is an effective and well-tolerated treatment option for patients with late presentation of HIV infection. No patient in this study discontinued therapy owing to adverse events, and the two-drug regimen demonstrated a favorable safety profile. However, lipid levels and body weight should be monitored regularly to enable timely intervention in the event of metabolic abnormalities.

## Data Availability

The raw data supporting the conclusions of this article will be made available by the authors, without undue reservation.

## Glossary

DTG + 3TC: dolutegravir plus lamivudine, including DTG/3TC (compound single tablet preparation) or DTG + 3TC (two pills) simplified double regimen
IQR: Interquartile range
CI: Confidence interval
NA: Not applicable
TND: Target not detected indicating below detection limit
NO: Not detected
AIDS: Acquired immunodeficiency syndrome
ART: Anti-retroviral therapy
rapid ART: ART initiated within 7 days of HIV-1 antibody confirmation
CVD: Cardiovascular disease
PJP: *Pneumocystis jirovecii* pneumonia
TB: Tuberculosis
BIC: Bictegravir
BMI: Body mass index
TC: Total cholesterol
TG: Triglycerides
HDL-C: High-density lipoprotein cholesterol
LDL-C: Low-density lipoprotein cholesterol
ALT: Alanine aminotransferase
LDH: Lactate dehydrogenase
TBIL: Total bilirubin
DBIL: Direct bilirubin
IBIL: Indirect bilirubin
Scr: Serum creatinine
eGFR: Estimated glomerular filtration rate
UA: Uric acid
AMY: Amylase
GLU: Glucose
